# Polysaccharide-Rich Red Algae (*Gelidium amansii*) Hot-Water Extracts Alleviate Abnormal Hepatic Lipid Metabolism without Suppression of Glucose Intolerance in a Streptozotocin/Nicotinamide-Induced Diabetic Rat Model

**DOI:** 10.3390/molecules27041447

**Published:** 2022-02-21

**Authors:** Shing-Hwa Liu, Chia-Yu Ku, Meng-Tsan Chiang

**Affiliations:** 1 Institute of Toxicology, College of Medicine, National Taiwan University, Taipei 10051, Taiwan; shinghwaliu@ntu.edu.tw; 2 Department of Medical Research, China Medical University Hospital, China Medical University, Taichung 40402, Taiwan; 3 Department of Pediatrics, College of Medicine, National Taiwan University Hospital, Taipei 10051, Taiwan; 4 Department of Food Science, National Taiwan Ocean University, Keelung 20224, Taiwan; shinpukenny@gmail.com

**Keywords:** red algae hot-water extracts, polysaccharide-rich, diabetic rats, glucose metabolism

## Abstract

This study was designed to investigate the effects of polysaccharide-rich red algae (*Gelidium amansii*) hot-water extracts (GHE) on lipid and glucose metabolism in rats with streptozotocin (STZ)/nicotinamide (NA)-induced diabetes. Rats were divided into three groups: NC—normal control group), DM—diabetic group, and DG—diabetic group supplemented with GHE (5%). The experimental diet and drinking water were available ad libitum for 10 weeks. After the 10-week feeding duration, the body weight, liver weight, total adipose tissue weight, and hepatic TBARS and cholesterol levels were significantly increased, and hepatic glycogen content and adipose lipolysis rate were significantly decreased in the DM group, which could be effectively reversed by supplementation of GHE. However, GHE supplementation could not improve the glucose intolerance in DM rats. It was interesting to note that GHE supplementation could decrease the liver glucose-6-phosphotase activity, which was increased in DM rats. Taken together, these results suggested that GHE feeding may ameliorate abnormal hepatic lipid metabolism, but not glucose intolerance, in diabetic rats induced by STZ/NA.

## 1. Introduction

Obesity and diabetes are the important health care issues in the world. Patients with type 2 diabetes have a higher risk of atherosclerosis, cerebrovascular disease, hypertension, hyperlipidemia, and other complications [1]. Clinical observations suggested that type 2 diabetes and obesity are closely linked [2,3,4,5]. Recent evidence suggested that algae have many beneficial effects on reducing plasma lipids, glucose, and obesity [6,7,8]. *Gelidium amansii* (GA) is an edible seaweed (red algae) that is widely distributed in Asian countries such as Korea, China, Japan, and Taiwan. The agar product (1,3-linked b-D-galactopyranose and 1,4-linked 3,6-anhydro-a-L-galactopyranose units) of GA [9] can be prepared to form a gel [10], which is a traditional food in Japan and Taiwan. GA, including both water-soluble components (e.g., water-soluble polysaccharides) and water-insoluble components (e.g., flavonoids), may have beneficial effects in the improvement of glucose and lipid metabolism. We have reported that supplementation of GA (algae) effectively ameliorated the impairment of glucose and lipid metabolism in a high-fructose diet/impaired glucose tolerance rat model [11] and a streptozotocin (STZ)/nicotinamide-induced diabetic rat model [12].

The water-soluble fibers in GA have been suggested to play a role in regulating lipid metabolism in a high-fat diet-induced hyperlipidemic hamster model [13]. The hot-water extracts of GA (GHE), an agar-filtered product rich in polysaccharides, can exert a downregulation effect on hepatic lipid metabolism through AMP-activated protein kinase (AMPK) phosphorylation and upregulation of peroxisome proliferator-activated receptor alpha (PPARα) and uncoupling protein-2 (UCP-2) in high-fat diet-induced obese hamsters [14]. GHE supplementation has been demonstrated to show beneficial effects on lipid metabolism in rats and hamsters fed a high-fat diet [13,14,15]. However, little information about the effect of GHE on glucose metabolism has been reported. We hypothesized that GHE supplementation would show beneficial effects not only on lipid metabolism, but also on glucose metabolism under a diabetic condition. In this study, we used a STZ/nicotinamide-induced diabetic rat model to investigate the effects of GHE feeding on blood glucose and insulin, glucose tolerance, hepatic glycogen and glycometabolism-related enzymes, and hepatic lipids in rats with diabetes.

## 2. Results and Discussion

GHE has been identified to contain moisture (6.5%), ash (4.6%), crude fat (0.25%), crude protein (6.7%), and nitrogen-free extract (81.95%) [13]. GHE contained 68.54% carbohydrate polymers, in which the major monosaccharide in water-soluble indigestible polysaccharides was galactose (86.0%) [14,15]. The polysaccharides in GHE, which have been analyzed by high-performance liquid chromatography (HPLC), contained three main major components with retention times of 6.00, 8.49, and 9.15 min, and the estimated molecule weights were 892, 26.5, and 10.5 kDa, respectively [15]. The 5% GHE has been demonstrated to improve the alteration of hepatic lipid homeostasis in high-fat diet-induced obese rats [15]. In the present study, we further investigated the effects of 5% GHE on glucose metabolism and hepatic lipid metabolism in a STZ/nicotinamide-induced diabetic rat model.

### 2.1. Changes in Body Weight, Food Intake, and Tissue Weight

**Figure 1:**
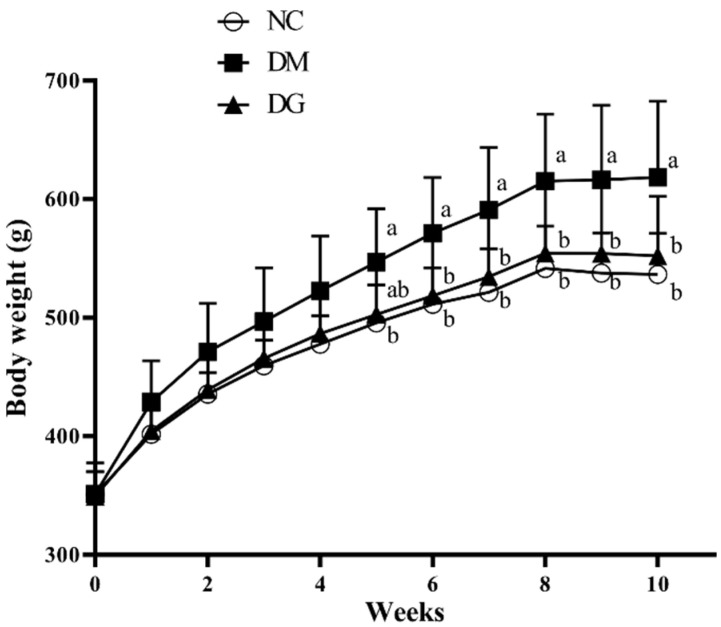
Effects of GHE supplementation on the body weight in rats with diabetes. Rats were fed different experimental diets for 10 weeks. Results are expressed as mean ± S.D. for each group (*n* = 7). Significant differences were determined by one-way ANOVA followed by Duncan’s multiple rand test. Different letters indicate statistical significance (*p* < 0.05). NC: normal control diet; DM: diabetic rats fed a high-fat diet; DG: diabetic rats fed a high-fat diet + 5% GHE.

Figure 1 shows the trend of body weight changes of rats during the experiment. There was a significant difference between the diabetic group (DM) and normal control group (NC) or diabetic group supplemented with GHE (5%) (DG) from the 5th week (*p* < 0.05) until the end of the experiment. As shown in Table 1, both final body weight and body weight gain in the DM group were significantly higher than in the NC or DG group (*p* < 0.05). During the experiment, the average food intake of the DG group was significantly lower than that of the DM group (*p* < 0.05), but there was no significant difference between NC and DM or DG (*p* < 0.05) (Table 1). In term of feed efficiency, both the DM and DG groups were significantly higher than the NC group (*p* < 0.05).

We next analyzed the changes in tissue weights. As shown in Table 2, both liver weight and liver weight/body weight ratio (relative liver weight) were significantly increased in the DM group compared to the NC group (*p* < 0.05), and this could be reversed by the supplementation of GHE (*p* < 0.05). The changes in adipose tissue weight are shown in Table 2. The weights of total adipose tissue, relative total adipose tissue, perirenal adipose tissue, and epididymal adipose tissue in the DM group were significantly higher than those in the NC group (*p* < 0.05), and this could be significantly reversed by the supplementation of GHE (*p* < 0.05). Moreover, in term of skeletal muscle weight, the relative weights of gastrocnemius muscle and soleus muscle in the DM group was significantly higher than that in both DM and DG groups (*p* < 0.05); there was no significant difference between NC and DG groups (*p* < 0.05) (Table 2). 

These results indicated that GHE supplementation could effectively improve the increased body weight and liver and adipose tissue weights, as well as the decreased muscle weight, in this diabetic rat model. The lower body weight due to GHE supplementation might have been related to the decreased adipose and liver tissue weights. The lower adipose tissue weight in diabetic rats by GHE supplementation might have been due to the increased adipose tissue hormone sensitive lipase (HSL) activity, increasing the lipolysis rate. Liu et al., reported that supplementation of GHE induced a significant increase in AMPK phosphorylation in rats fed a high-fat diet [15]. It is possible that the induced HSL activity by GHE supplementation may be related to the increased AMPK phosphorylation, because the increased AMPK phosphorylation can promote HSL activation in adipose tissue [16]. Another possible reason for lower body weights due to GHE was that there was lower food intake in diabetic rats fed with the GHE supplementation diet, because water-soluble dietary fibers in GHE can increase satiety, suppress appetite, and delay gastric emptying [17].

### 2.2. Changes in Blood Glucose and Insulin Levels, Glucose Tolerance, Hepatic Lipids, Glycogen Content, Glycometabolism-Related Enzymes, TBARS, and Adipose Tissue Lipolysis Rate

**Figure 2:**
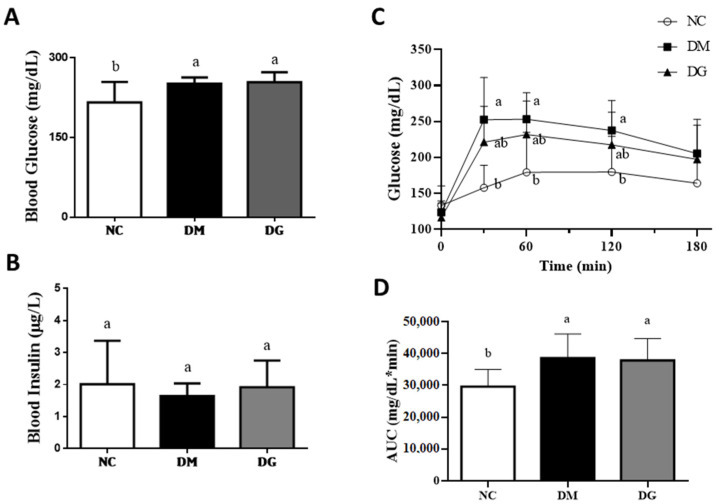
Effects of GHE supplementation on the blood glucose and insulin levels and glucose tolerance (OGTT) in rats with diabetes. Rats were fed different experimental diets for 10 weeks. The levels of blood glucose (**A**) and insulin (**B**) levels are shown. The OGTT (**C**) and area under the curve (AUC) (**D**) are shown. Results are expressed as mean ± S.D. for each group (*n* = 7). Significant differences were determined by one-way ANOVA followed by Duncan’s multiple rand test. Different letters indicate statistical significance (*p* < 0.05). NC: normal control diet; DM: diabetic rats fed a high-fat diet; DG: diabetic rats fed a high-fat diet + 5% GHE.

It has been shown that long-term intake of water-soluble fiber helps to improve the hyperinsulinemia and hyperlipidemia of type 2 diabetes [18]. We next tested the effects of GHE supplementation on circulating glucose regulation in a diabetic rat model. As shown in Figure 2A, the blood glucose concentrations in both the DM and DG groups were significantly higher than that in the NC group (*p* < 0.05), while there was no significant difference between the DM group and the DG group (*p* > 0.05). For the plasma insulin test, there were no significant differences among these three groups (*p* > 0.05) (Figure 2B). Figure 2C shows the changes in blood glucose in an oral glucose tolerance test (OGTT) of rats with or without diabetes. At the 30th, 60th, and 120th minutes, the blood glucose concentrations in DM group were significantly higher than those in the NC group (*p* < 0.05), while the blood glucose concentrations in the DG group were not significantly different from that of the DM group (*p* > 0.05). Figure 2D shows the changes in the area under the curve (AUC) of the OGTT. The AUC in both the DM group and DG group were significantly higher than those in the NC group (*p* < 0.05). There was no significant difference between the DM and DG groups (*p* > 0.05).

**Figure 3:**
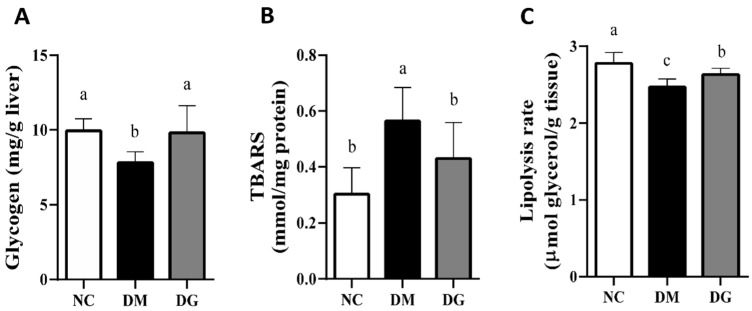
Effects of GHE supplementation on the hepatic glycogen and TBARS and adipose lipolysis rate in rats with diabetes. Rats were fed different experimental diets for 10 weeks. (**A**) The levels of hepatic glycogen are shown. (**B**) The hepatic TBARS levels are shown. (**C**) The lipolysis rate in perirenal adipose tissue is shown. Results are expressed as mean ± S.D. for each group (*n* = 7). Significant differences were determined by one-way ANOVA followed by Duncan’s multiple rand test. Different letters indicate statistical significance (*p* < 0.05). NC: normal control diet; DM: diabetic rats fed a high-fat diet; DG: diabetic rats fed a high-fat diet + 5% GHE.

We next analyzed the effects of GHE on lipids, lipid peroxidation, and glycometabolism-related molecules in the liver and the lipolysis rate in the adipose tissue. As shown in Table 3, the triglyceride and total cholesterol concentrations in the livers of the DM group were significantly higher than those in the NC group (*p* < 0.05), and this could be significantly reversed by the supplementation of GHE (*p* < 0.05). As shown in Table 4, there was no significant difference in the liver hexokinase activity among these three groups (*p* > 0.05). The liver glucose-6-phosphatase activity and glucose-6-phosphatase/hexokinase ratio in the DM group were significantly higher than in both the NC and DG groups (*p* < 0.05); there was no significant difference between the DG and NC groups (*p* > 0.05). Figure 3A shows the change in the liver glycogen content. The hepatic glycogen contents in the DM group were significantly lower than those in the NC and DG groups (*p* < 0.05), but there was no significant difference between the DG and NC groups (*p* > 0.05). Figure 3B shows the change in the liver TBARS level. The hepatic thiobarbituric acid reactive substances (TBARS) concentrations in the DM group were significantly higher than those in both the NC and DG groups (*p* < 0.05); there was no significant difference between the DG and NC groups (*p* > 0.05). Moreover, the lipolysis rate in the perirenal adipose tissue of the DM group was significantly lower than those of both the NC and DG groups (*p* < 0.05), while there was a significant difference between the NC and DG groups (*p* < 0.05) (Figure 3C).

GA (red algae), including both water-soluble components (e.g., water-soluble polysaccharides) and water-insoluble components (e.g., flavonoids), may have beneficial effects on the improvement of lipid and glucose metabolism. Our previous work showed that GA might improve plasma glucose and lipids and increase lipolysis activity, resulting in reduced adipose tissue, thereby reducing tumor necrosis factor (TNF)-α, interleukin (IL)-6, and plasminogen activator inhibitor (PAI)-1 and a lowered risk of chronic inflammation and cardiovascular disease in rats with diabetes [12]. Supplementation of GHE in the diet has been found to induce the activation of AMPK, increase farnesoid-X receptor (FXR) and PPARα protein expression, and decrease PPAR-γ protein expression to inhibit lipogenic enzyme activities and reduce the lipid accumulation in the livers of rats fed on a high-fat diet [15]. In addition, GHE could reduce the expression of hepatic sterol regulatory element-binding proteins (SREBPs) [13], and activated AMPK signaling [13,14] in the livers of high-fat diet-induced obese hamsters, thereby decreasing the hepatic lipogenesis. In the present study, we found that GHE supplementation in the diet possessed a similar ability to alleviate the abnormal hepatic lipids and adipose lipolysis rate in rats with diabetes.

Some potentially regulatory pathways are involved in glucose and lipid metabolism during diabetic conditions. The upregulation of mitogen-activated protein kinase (MAPK; p38, ERK, JNK) expression has been found in the livers of STZ/nicotinamide-induced diabetic rats that can be inhibited by Eugenosedin-A, which can improve glucose metabolism [19]. The polyphenol extract of *Caesalpinia bonduc* has been shown to ameliorate hyperglycemia in diabetic rats through mechanisms including reactive oxygen species (ROS) scavenging, reducing lipid peroxidation, increasing antioxidant enzyme activity, upregulating insulin secretion, and inhibiting the MAPK-JNK signaling pathway, which contributes to β-cell apoptosis [20]. The downregulation of glucose transport activity, such as glucose transporter (GLUT)2 in the liver and GLUT4 in the skeletal muscle, is known to be associated with insulin resistance and type 2 diabetes [21]. The protein expression of GLUT4 has been found to be downregulated in the skeletal muscle of STZ/nicotinamide-induced diabetic rats [19]. Inhibitors of apoptosis (IAPs) are a family of proteins that function as intrinsic regulators of the caspase cascade [22]. Cellular IAP-1 has been demonstrated to play an important role in pancreatic β-cell survival [23]. In an in vivo allogeneic islet graft study, the overexpression of X-linked IAP was shown to enhance the survival of islet transplants in diabetic mice [24]. On the other hand, gut microbiome dysbiosis is known as one of the critical factors in metabolic diseases such as diabetes and obesity. The dietary polysaccharides from plants and food sources have been suggested to be the positive modulators of the gut microbiome [25]. Therefore, the effects of supplementation of GHE on the above regulatory pathways and gut microbiome in STZ/nicotinamide-induced diabetic rats may be worth further investigation in the future.

GA can improve the impairment of glucose and lipid metabolism in a high-fructose diet-fed rat model [11]. In the present study, we found that GHE induced a significant decrease in hepatic glucose phosphatase activity and the TBARS value, and an increase in hepatic glycogen content in rats with diabetes, indicating that GHE may reduce hepatic gluconeogenesis. However, there were no significant changes in OGTT and plasma glucose and insulin levels in diabetic rats fed a diet with GHE supplementation. It was reported by Kim et al., that red algae contain bromophenols, such as 2,4,6-tribromophenol and 2,4-dibromophenol, which may reduce α-glucosidases and moderately regulate glucose metabolism [26]. Polysaccharide-rich GHE may contain few bromophenols because tribromophenol is only slightly soluble. Therefore, GA can ameliorate insulin resistance, but its hot-water extract GHE does not possess this ability. Two explanations can be proposed: (a) GHE feeding duration is insufficient in diabetic animals; and (b) the insoluble matters of functional ingredients for lowering blood glucose in GA, such as bromophenols and some flavonoids, were removed during the production of GHE. Nevertheless, further study is required to identify the insoluble parts of GA.

The supplementation of functional foods or natural bioactive compounds have been found to contribute to significant improvements in body weight, glucose and lipid metabolism, endothelial damage, blood pressure, inflammation, and oxidative stress in clinical management of metabolic syndromes [27]. A combination of nutraceuticals that contained *Lagerstroemia speciosa*, *Berberis aristata*, *Curcuma longa*, α-lipoic acid, chromium picolinate, and folic acid was shown to have clinically beneficial effects on glucose and lipid metabolism in patients with impaired fasting glucose [28]. In the present study, we found that GHE supplementation could reduce body weight, liver and adipose tissue weights, and hepatic lipids and TBARS; enhance hepatic glycogen content; and decrease glucose-6-phosphatase activity in diabetic rats. Therefore, nutraceuticals and/or antioxidants/anti-inflammatory compounds are being considered as a definite therapeutic strategy in the treatment of glucose and lipid metabolism disorders during diabetic conditions.

## 3. Materials and Methods

### 3.1. Animals and Experimental Diets

Seven-week-old male Sprague Dawley (SD) rats purchased from BioLASCO (Taipei, Taiwan) were housed in the animal room of the Animal Experiment Center, National Taiwan Ocean University. Rats were acclimated for one week and fed a chow diet (Laboratory Rodent Diet 5001, LabDiet, St. Louis, MO, USA). The animal room was maintained at 23 ± 1 °C and 40–60% humidity with a daily 12-hour light/dark cycle. Both feed and water were provided ad libitum. Body weight and food intake were measured once a week. The experimental procedures were approved by the Animal House Management Committee of the National Taiwan Ocean University. The experiments were conducted according to the ARRIVE guidelines for care and use of laboratory animals.

Rats were randomly divided into three groups (7 rats in each group): rats fed a normal chow diet (NC group,), diabetic rats fed a high-fat diet (DM group), and diabetic rats fed a high-fat diet with 5% GHE (DG group). The preparation and composition of the diets were conducted as previously described [15]. Briefly, the compositions of the diets were as follows: for the NC group—100% chow diet; for the DM group—89.30% chow diet + 10% lard and others; for the DG group—83.30% chow diet + 11% lard + 5% GHE and others. The total calories (kcal/100 g) in the diets for these three groups were 336.20, 394.73, and 394.54, respectively. The experiment was carried out for a total of 10 weeks. For induction of diabetes, rats were subcutaneously given nicotinamide (NA; Sigma, St. Louis, MO, USA) with 230 mg/kg body weight and streptozotocin (STZ; in 0.05 M citrate buffer; pH 4.5; Sigma, St. Louis, MO, USA) with 65 mg/kg body weight as previously described [12]. One week later, fasting rats were subjected to a test of blood glucose levels after a 2 h oral glucose tolerance test (OGTT). The diabetic condition was confirmed when the blood glucose levels were more than 200 mg/dL within 2 h after the glucose challenge.

The polysaccharide-rich hot-water extract of GA (GHE) was prepared as previously described [13,15]. In brief, dry material of GA, which was purchased from the market at Keelung, Taiwan, was added into deionized water and heated at 121 °C for 20 min. The recovery rate of the GHE was about 43%. The solution was then cooled, filtered, and lyophilized. In this study, we used 5% GHE to test its effects on altered glucose metabolism and hepatic lipids in a diabetic rat model. The dosage of 5% was selected according to the study of Liu et al. [15].

### 3.2. Blood Glucose and OGTT Assay

For OGTT, fasting rats were orally administered with glucose (2 g/kg body weight), and tail vein blood samples were collected at 0, 30, 60, 90, 120, and 180 min. Plasma levels of glucose were detected with an enzymatic kit (RANDOX Laboratories, Antrim, UK). 

### 3.3. Blood and Tissue Collection

At the end of the experiment, rats were euthanized under anesthesia, and blood samples were collected from the abdominal aorta and then centrifuged at 3000 rpm for 20 min. The supernatants were collected as the plasma to be analyzed. Tissue samples of the liver, adipose, and small intestine were collected, weighed, and stored at −80 °C until analysis.

### 3.4. Liver Lipid Measurement

The liver extractions were determined as previously described [29]. Briefly, 0.2 g of liver tissue in 4 mL of mixed solution (chloroform: methanol = 2:1, *v*/*v*) were homogenized with a tissue homogenizer under an ice bath, and then centrifuged at 3000 rpm at 4 °C for 10 min. The supernatants were stored at −20 °C until analysis. The levels of TG and TC in the liver were measured as previously described [30]. Briefly, 10 μL of liver tissue extracts were mixed with 10 μL Triton X-100 in a glass test tube, and then placed in a refrigerated vapor trap to remove the organic solvents. The liver total cholesterol and triglyceride contents were determined with enzymatic kits (RANDOX Laboratories).

### 3.5. Hepatic Thiobarbituric Acid Reactive Substances (TBARS) Determination

The hepatic TBARS level was determined as previously described [31]. Briefly, the liver samples in the KCl solution were homogenized, and then centrifuged at 3000 rpm at 4 °C for 10 min. The supernatants were then collected. Samples or standard (1,1,3,3-tetraethoxypropane; Sigma-Aldrich, St. Louis, MO, USA) were added into a solution containing 1% H_3_PO_4_ and 0.67% thiobarbituric acid (TBA), and then heated at 95 °C for 45 min. After cooling, the reactive solution was mixed with n-butanol and centrifuged at 3000 rpm at 4 °C for 10 min. The absorbances at 535 nm and 520 nm were detected using an ELISA Microplate Reader (BioTek, Winooski, VT, USA).

### 3.6. Lipolysis Rate Analysis

The lipolysis rate was detected as previously described [32]. Briefly, the minced adipose tissues were reacted in a working buffer containing N-tris-(hydroxymethyl)methyl-2-aminoethanesulfonic acid buffer (pH 7.4) and isoproterenol at 37 °C. After reaction, the product of glycerol was analyzed with a glycerol assay kit (Randox Laboratories, London, UK), and the absorbance at 520 nm was detected. The lipolysis rate is presented as μmol glycerol release per gram tissue per hour.

### 3.7. Glycogen Content and Measurement of Activities of Hexokinase, Glucose-6-Phosphatase (G6Pase), and Glucose-6-Phosphate Dehydrogenase (G6PD) 

The glycogen content was determined as previously described [33]. Briefly, the liver samples were homogenized with citrate buffer (pH 4.2), and then centrifuged at 10,600× *g* 4 °C for 30 min. The supernatants were reacted with amyloglucosidase (Sigma-Aldrich) to release glucose. The glucose content was analyzed with a glucose assay kit (Audit Diagnostics, Cork, Ireland).

The liver tissue extracts were prepared according to the method of Nagayama et al., (1972) [34] with a modification. Briefly, liver tissues in N-acetyl-cysteine buffer (pH 7.0) were homogenized under an ice bath and centrifuged at 15,000 rpm at 4 °C for 40 min to obtain the cytosol fraction. The hexokinase activity was detected as previously described [35]. Briefly, the liver cytosol fraction was added to the solution containing reagents with 0.25 M glycylglycine buffer (pH 7.5), 0.75 M MgSO_4_, 7.5 mM NADP, 0.75 M glucose, 1 M KCl, 0.75 M ATP, and 7 unit/mL G6PD for reaction. The reaction generated the gluconate-6-phosphate and reduced NADP to NADPH. The absorbance at 340 nm was determined by a microplate reader. The hexokinase activity is presented as nmol NADPH/min/mg protein.

The principle for G6Pase activity analysis was based on the fact that glucose-6-phosphate (G6P) can be catalyzed by G6Pase to generate inorganic phosphate. The liver cytosol fraction was added to the solution containing reagents with 0.05 M Tris-maleate buffer (pH 7.5) and 0.1 M G6P for reaction. The reaction generated the inorganic phosphorus. The inorganic phosphorus was further detected using a microcolorimetric method as previously described [36]. The reaction solution and 5% trichloroacetic acid were mixed and centrifuged at 3000 rpm at 4 °C for 30 min to collect the supernatants, in which the final reagents with H_2_SO_4_ (5 N), 2.5% ammonium molybdate, and 10% ascorbic acid were added for reaction. The absorbance at 660 nm was analyzed by a microplate reader. The G6Pase activity is presented as nmol Pi/min/mg protein.

### 3.8. Statistical Analysis

The experimental data were analyzed using IBM SPSS 22.0 statistical analysis software. Data are presented as mean ± standard deviation (S.D.). The one-way ANOVA and post-hoc Duncan’s multiple range test were used. If *p* < 0.05, there was a significant difference between groups.

## 4. Conclusions

**Figure 4:**
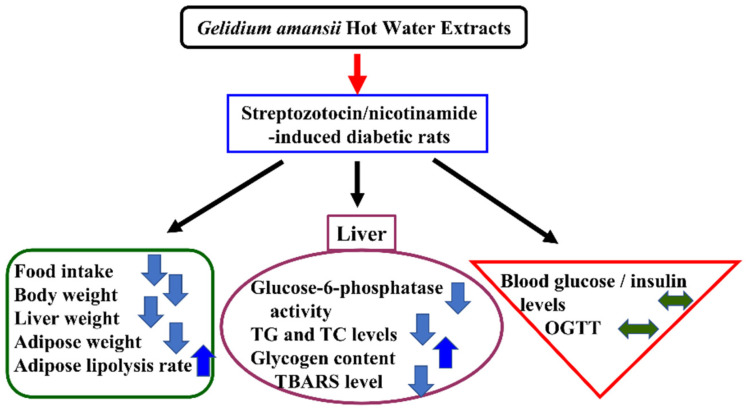
Scheme representing the effects of polysaccharide-rich red algae (*Gelidium amansii*) hot-water extracts on lipid and glucose metabolism in streptozotocin/nicotinamide-induced diabetic rats. OGTT: oral glucose tolerance test; TBARS: thiobarbituric acid reactive substances; TC: total cholesterol; TG: triglyceride.

The beneficial effects of GHE on lipid metabolism have been demonstrated in high-fat diet-induced obese animal models [13,14,15]. However, to date, no beneficial effects of GHE on glucose metabolism in STZ/nicotinamide-induced diabetic animal models have been established. In the present study, we demonstrated that GHE supplementation significantly ameliorated the altered hepatic lipids, but had a limited ability to improve the impairment of glucose homeostasis in rats with diabetes. GHE supplementation decreased body weight, liver and adipose tissue weights, and hepatic lipids and TBARS in diabetic rats. In addition, diabetic rats fed with GHE had higher hepatic glycogen content and lower glucose-6-phosphatase activity, indicating that GHE may ameliorate hepatic gluconeogenesis. However, there were no significant changes in OGTT or blood glucose and insulin levels after GHE treatment. These results are summarized in Figure 4. The water-insoluble components in GA, which contain the regulatory function in blood glucose, may require further investigation in the future.

## Figures and Tables

**Table 1:** The effects of GHE on body weight, food intake, and food efficiency in rats fed with different experiment diets for 10 weeks.

Diet	NC	DM	DG
Initial body weight (g)	351.0 ± 19.0 ^a^	351.2 ± 26.3 ^a^	351.3 ± 20.6 ^a^
Final body weight (g)	500.8 ± 30.0 ^b^	591.0 ± 57.7 ^a^	530.0 ± 50.6 ^b^
Body weight gain (g)	160.4 ± 20.3 ^c^	232.2 ± 35.0 ^a^	187.5 ± 36.0 ^bc^
Food intake (g/day)	29.8 ± 1.99 ^ab^	30.5 ± 2.43 ^a^	27.6 ± 2.12 ^b^
Feed efficiency (%)	5.38 ± 0.43 ^b^	7.61 ± 0.92 ^a^	6.79 ± 1.22 ^a^

Results are expressed as mean ± S.D. for each group (*n* = 7). Significant differences were determined by one-way ANOVA followed by Duncan’s multiple rand test. Different letters indicate statistical significance (*p* < 0.05). NC: normal control diet; DM: diabetic rats fed a high-fat diet; DG: diabetic rats fed a high-fat diet + 5% GHE.

**Table 2:** The effects of GHE on tissue/organ weights in rats fed with different experimental diets for 10 weeks.

Diet	NC	DM	DG
Liver weight (g)	13.3 ± 0.92 ^c^	30.7 ± 4.94 ^a^	24.0 ± 3.24 ^b^
Relative liver weight (g/100 g B.W.)	2.62 ± 0.12 ^c^	5.18 ± 0.58 ^a^	4.53 ± 0.39 ^b^
Total adipose tissue weight (g)	12.9 ± 2.81 ^b^	20.6 ± 5.30 ^a^	14.2 ± 4.17 ^b^
Relative adipose tissue weight (g/100 g B.W.)	2.31 ± 0.43 ^b^	3.47 ± 0.76 ^a^	2.66 ± 0.67 ^b^
Perirenal adipose weight (g)	5.95 ± 1.70 ^b^	10.9 ± 2.49 ^a^	7.39 ± 2.29 ^b^
Relative perirenal adipose weight (g/100 g B.W.)	1.16 ± 0.29 ^b^	1.83 ± 0.37 ^a^	1.38 ± 0.37^ab^
Epididymal adipose weight (g)	5.91 ± 1.44 ^b^	9.76 ± 2.88 ^a^	6.80 ± 2.19 ^b^
Relative epididymal adipose weight (g/100 g B.W.)	1.15 ± 0.22 ^b^	1.63 ± 0.41 ^a^	1.28 ± 0.37 ^ab^
Gastrocnemius muscle weight (g)	6.18 ± 0.40 ^a^	6.08 ± 0.51 ^a^	6.03 ± 0.53 ^a^
Relative gastrocnemius muscle weight (g/100 g B.W.)	1.21 ± 0.08 ^a^	1.03 ± 0.06 ^c^	1.14 ± 0.10 ^ab^
Soleus muscle weight (g)	0.41 ± 0.08 ^a^	0.44 ± 0.07 ^a^	0.42 ± 0.07 ^a^
Relative soleus muscle weight (g/100 g B.W.)	0.08 ± 0.01 ^a^	0.07 ± 0.01 ^b^	0.08 ± 0.01 ^a^

Results are expressed as mean ± S.D. for each group (*n* = 7). Significant differences were determined by one-way ANOVA followed by Duncan’s multiple rand test. Different letters indicate statistical significance (*p* < 0.05). NC: normal control diet; DM: diabetic rats fed a high-fat diet; DG: diabetic rats fed a high-fat diet + 5% GHE.

**Table 3:** The effects of GHE on hepatic lipid levels in rats fed with different experimental diets for 10 weeks.

Diet	NC	DM	DG
Triglyceride			
(mg/g liver)	12.5 ± 5.58 ^b^	67.6 ± 31.7 ^a^	56.1 ± 20.6 ^a^
(g/liver)	0.17 ± 0.08 ^c^	2.13 ± 1.23 ^a^	1.32 ± 0.42 ^b^
Total cholesterol			
(mg/g liver)	3.67 ± 1.42 ^c^	96.3 ± 11.6 ^a^	81.2 ± 20.9 ^b^
(g/liver)	0.05 ± 0.02 ^c^	2.98 ± 0.64 ^a^	1.96 ± 0.60 ^b^

Results are expressed as mean ± S.D. for each group (*n* = 7). Significant differences were determined by one-way ANOVA followed by Duncan’s multiple rand test. Different letters indicate statistical significance (*p* < 0.05). NC: normal control diet; DM: diabetic rats fed a high-fat diet; DG: diabetic rats fed a high-fat diet + 5% GHE.

**Table 4:** The effects of GHE on hepatic enzyme activities in rats fed with different experimental diets for 10 weeks.

Diet	NC	DM	DG
Hexokinase (nmol/min/mg protein)	5.96 ± 1.48 ^a^	4.44 ± 1.33 ^a^	5.94 ± 3.12 ^a^
Glucose-6-phosphatase (nmol/min/mg protein)	0.32 ± 0.03 ^c^	0.5 ± 0.07 ^a^	0.44 ± 0.04 ^b^
Glucose-6-phosphatase /Hexokinase ratio	0.06 ± 0.02 ^c^	0.12 ± 0.03 ^a^	0.09 ± 0.03 ^b^

Results are expressed as mean ± S.D. for each group (*n* = 7). Significant differences were determined by one-way ANOVA followed by Duncan’s multiple rand test. Different letters indicate statistical significance (*p* < 0.05). NC: normal control diet; DM: diabetic rats fed a high-fat diet; DG: diabetic rats fed a high-fat diet + 5% GHE.

## Data Availability

The data presented in this study are available from the corresponding author upon reasonable request.

## Sample Availability

Samples of the compounds are not available from the authors.
